# Squalene epoxidase, located on chromosome 8q24.1, is upregulated in 8q+ breast cancer and indicates poor clinical outcome in stage I and II disease

**DOI:** 10.1038/sj.bjc.6604556

**Published:** 2008-08-12

**Authors:** M W Helms, D Kemming, H Pospisil, U Vogt, H Buerger, E Korsching, C Liedtke, C M Schlotter, A Wang, S Y Chan, B H Brandt

**Affiliations:** 1 Department of Pediatrics, Stanford University School of Medicine, Stanford, CA, USA; 2 UKE Hamburg, Institute for Tumor Biology, Hamburg, Germany; 3 Center for Bioinformatics, University of Hamburg, Hamburg, Germany; 4 European Laboratory Association, Ibbenbueren/Osnabrück, Germany; 5 Institute of Pathology, University of Muenster, Muenster, Germany; 6 Institute of Gynecology and Obstetrics, University of Muenster, Muenster, Germany; 7 Department of Breast Medical Oncology, The University of Texas MD Anderson Cancer Center, Houston, TX, USA; 8 Department of Gynecology and Obstetrics, Breast Centre, Academic Hospital of University Bonn, Klinikum Lüdenscheid, Lüdenscheid, Germany; 9 Celera Diagnostics, Alameda, CA, USA

**Keywords:** squalene epoxidase, oestrogen receptor, LIV-1, breast cancer, 8q, 7p

## Abstract

Gains of chromosomes 7p and 8q are associated with poor prognosis among oestrogen receptor-positive (ER+) stage I/II breast cancer. To identify transcriptional changes associated with this breast cancer subtype, we applied suppression subtractive hybridisation method to analyse differentially expressed genes among six breast tumours with and without chromosomal 7p and 8q gains. Identified mRNAs were validated by real-time RT–PCR in tissue samples obtained from 186 patients with stage I/II breast cancer. Advanced statistical methods were applied to identify associations of mRNA expression with distant metastasis-free survival (DMFS). mRNA expression of the key enzyme of cholesterol biosynthesis, squalene epoxidase (SQLE, chromosomal location 8q24.1), was associated with ER+ 7p+/8q+ breast cancer. Distant metastasis-free survival in stage I/II breast cancer cases was significantly inversely related to SQLE mRNA in multivariate Cox analysis (*P*<0.001) in two independent patient cohorts of 160 patients each. The clinically favourable group associated with a low SQLE mRNA expression could be further divided by mRNA expression levels of the oestrogen-regulated zinc transporter LIV-1. The data strongly support that SQLE mRNA expression might indicate high-risk ER+ stage I/II breast cancers. Further studies on tumour tissue from standardised treated patients, for example with tamoxifen, may validate the role of SQLE as a novel diagnostic parameter for ER+ early stage breast cancers.

Distinct chromosomal copy number gains are common in breast cancer and indicate distinct genetic pathways in tumorigenesis. In systematic studies, using comparative genomic hybridisation (CGH), chromosomal gains were observed most commonly on chromosomes 1q (50–55%), 8q (60%), 16p (40–60%), 17q (25–30%) and 20q (20–25%) (Rennstam *et al*, 2003; Fridlyand *et al*, 2006; Mitelman *et al*, 2007). A particularly poor clinical outcome has been associated with 8q gains (Rennstam *et al*, 2003). Moreover, we have previously characterised a subtype of stage I/IIa oestrogen receptor-positive (ER+) poorly differentiated breast carcinomas displaying gains of chromosome 7p. These tumours exhibit shorter time to progression compared with other breast cancer phenotypes and high frequency of 8q gains (Rennstam *et al*, 2003; Korsching *et al*, 2004; Helms *et al*, 2005). In addition, a high proliferation rate combined with a low HER2, p53 and EGFR expression and a high degree of cytogenetic instability were found among these tumours. A more precise definition of these patients might determine which of these ER+ node-negative breast cancer patients would benefit from additional treatment besides endocrine therapy.

Recent studies have provided solid evidence that tumour gene expression profiles can provide useful prognostic information for patients with both node-negative and node-positive breast cancer (O'Shaughnessy, 2006; Massague, 2007; Mook *et al*, 2007).

Therefore, to identify novel markers useful for gene expression-based patient prognosis, we compared mRNA expression of breast cancer cases displaying gains of 7p and 8q with cases showing no aberrations on chromosomes 7p and 8q by suppression subtractive hybridisation PCR (SSH). Differential expression of 60 gene products identified by SSH was first validated by real-time RT–PCR using the original sample set. A correlation analysis between 12 validated differentially regulated genes and (i) clinical parameters and (ii) established molecular parameters (i.e. ER, EGFR, HER2, p53 and Bcl2) on a cohort of 160 stage I/II patients with full clinical records and long-term follow-up was performed to assess prognostic relevance.

Using the exploratory approach of recursive partitioning and regression tree analysis, squalene epoxidase (SQLE) was identified as the most significant parameter to cluster patients by outcome. An additional impact was shown for the ER-related gene LIV-1 by further splitting the SQLE-low subgroup of patients. These results held true in multivariate Cox analysis when compared to established risk factors in stage I/II breast cancer.

## Materials and methods

### Suppression subtractive hybridisation

The study protocol for the storage, processing and analysis of patient material was approved by the local ethics committee.

Suppression subtractive hybridisation expression analysis was performed on six ductal invasive grade 2 and 3 breast cancer cases with very similar protein expression pattern (ER-positive, PR-positive, erbB2 0–2, Dako-Score/no-erbB2-amplification, EGFR-negative, p53 0–1, Ck 5-negative, Ck 8/18-positive) and cytogenetic background except for 7p and 8q (all tumours displayed a loss of chromosome 16q), which has been previously described in more detail (Korsching *et al*, 2004). Three of the six tumours harboured a gain on chromosome 7p and 8q (designated as ‘gain 7/8’) as opposed to three cases without these cytogenetic changes (norm 7/8).

The gain 7/8 tumours displayed more than 20% Mib-1 staining and more than 12 genetic alterations on average per case as revealed by CGH analysis. In contrast, norm 7/8 tumours exhibited only 5–10% Mib-1 staining and 5.3 genetic alterations on average per case.

Five 10-*μ*m sections of morphological homogenous tumour areas were pooled from fresh frozen tissue and total RNA was isolated by StrataPrep total RNA Microprep Kit (Stratagene, Amsterdam, The Netherlands) including DNase digestion. cDNA was synthesised and amplified to about 3 *μ*g cDNA starting from 100 ng total RNA derived from each sample with the SMART PCR cDNA Synthesis Kit (BD Clontech, Heidelberg, Germany).

Suppression subtractive hybridisation was performed with the PCR-Select cDNA Subtraction Kit (BD Clontech). To equalise the cytogenetic background, cDNA from three specimens were pooled for each group and analysed by SSH in forward and reverse directions.

### Creation of a subtracted cDNA library

Freshly prepared 10 cycle secondary SSH-PCRs resulting from the forward and the reverse subtractions, respectively, were cloned into the pCR 2.1. vector using the Original T/A Cloning Kit (Invitrogen, Karlsruhe, Germany) and transformed to *Escherichia coli* TOP 10 F' according to the manufacturer's instructions.

### Sequencing of subtracted cDNA library

One hundred and ninety-two bacterial clones resulting from each of the subtractions were picked and cultured in 15 ml TB medium in deep-well plates at 37°C overnight. Plasmids were isolated using the Wizard Magnesil Plasmid Purification System (Promega, Mannheim, Germany) and the inserts sequenced with a M13 forward Promotor (−21) primer (5′-TGTAAAACGACGGCCAGT-3′) using standard techniques. The sequences of the inserts of differentially expressed genes were identified using NCBI Blast search (blastn)(Altschul *et al*, 1997).

### Relative expression quantification of SSH hits by real-time RT–PCR

Expression of 60 of the genes identified as differentially expressed by SSH was quantified in all the six tumour specimens by SYBR Green real-time RT–PCR. For this purpose, DNase-treated total RNA derived from the six tumours was reverse transcribed using Multiscribe Reverse Transcriptase and random hexamers (Applied Biosystems, Foster City, CA, USA) according to the manufacturer's instructions. Quantitative PCR analyses were performed on an ABI 7900 Sequence Detection System (Applied Biosystems) in duplexes using the Quantitect SYBR Green PCR kit (Qiagen, Hilden, Germany) and normalised to 18S rRNA (Pre-Developed TaqMan Assay Reagents Control Kit; Applied Biosystems). Primer design was performed with the help of the ‘Primer Express’ software (Applied Biosystems). To prevent the amplification of genomic sequences, at least one primer of each pair was designed spanning two exons. Specificity of amplification was checked by melting curve analysis. Primer oligonucleotides were purchased from Operon (Cologne, Germany). PCR conditions were: initial denaturation for 10 min at 95°C followed by 50 cycles consisting of 15 s at 95°C and 1 min at 60°C. Expression ratios between two samples were calculated from differences in threshold cycles at which an exponential increase in reporter fluorescence could first be detected (*C*_T_ values). Results of duplicate experiments were averaged.

### Real-time RT–PCR profiling on large breast cancer sample set

In addition to the six samples used for SSH, a fresh frozen breast tumour sample set accrued from 1986 to 1998 at the University Medical Centre Muenster served as the source of samples for the prognostic evaluation of differentially expressed genes. The inclusion criteria for the primary study included: T1 or T2 tumours, lymph node-negative (determined by full axillary lymph node dissection) and follow-up for 5 years. 186 patients had known recurrence status at the last follow-up. A subset of 160 patients (of 186) was evaluable for clinical follow-up that has been classified for ER, PR, HER2 and therapy. We chose distant metastasis-free survival (DMFS) as the primary end point because it is most directly linked to cancer-related death and cancer cell motility.

### Sample processing

Total RNA was isolated from 50 to 100 mg of frozen breast cancer tissues using RNeasy Mini kit (Qiagen, Valencia, CA, USA). To ensure absence of DNA contamination, an on-column DNase I treatment was performed. Total RNA was quantified using spectrophotometer OD260 measurements. The amplifiable RNA was determined by measuring the expression level of a housekeeping gene (EEF1A1).

### Gene expression profiling

One-step RT–PCR with SYBR® Green was used for gene expression profiling as published previously (Rogge *et al*, 2000). Pre-developed and validated PCR primers (Applied Biosystems) were used to amplify all known splice variants. Fifteen-microlitre reactions were performed in duplicates using amplification plates in 384-well format. The cycle profile consists of 50°C 2 min, 95°C 1 min, 60°C 30 min, followed by 45 cycles of 95°C 15 s and 60°C 30 s ending with dissociation analysis. Together with three normalisation genes, the expression levels of a total of 59 targets in 186 RNA samples were profiled. The expression level of each gene was normalised with the average of expression levels of three housekeeping genes (NUP214, PPIG and SLU7). The normalised expression levels of 59 mRNAs were used for statistical analyses.

### Statistical analysis

Distant metastasis-free survival was chosen as the primary end point of this study. Distant metastasis-free survival is defined as the interval between the date of definitive breast cancer surgery and diagnosis of first distant metastasis or the last date of follow-up (whichever occurred first). Contralateral recurrences and deaths without recurrence were regarded as censoring events, whereas local recurrences were considered neither as events nor as censoring events. The definition of DMFS end points, its events and censoring rules were aligned with those adopted by the National Surgical Adjuvant Breast and Bowel Project for the prognostic molecular marker studies (Paik *et al*, 2004).

Disease-free survival was defined as the interval between the date of disease diagnosis and diagnosis of the first local, regional or distant tumour recurrence, patient death or date of last follow-up. Overall survival (OS) was calculated as the interval between disease diagnosis and patient death (regardless of the cause).

Regression trees were built through an iterative process called binary recursive partitioning, which comprises the splitting of the data into partitions and further splitting of the branches to minimise the squared deviations. Kaplan–Meier survival estimates were generated and compared with the log-rank test. Multivariate analysis was performed using Cox proportional hazards regression model. Level of significance was *P*=0.05. Cox proportional hazards regression models have been computed using the Efron approximation from the ‘survival’ package within the statistical data analysis environment R version 2.5.0 (R Development Core Team, 2007).

### Analysis of microarray data

The publicly available data set of Loi *et al* (2007) was used for correlation analyses of SQLE expression and survival. The cel files (GEO accession no. GSE6532) were downloaded from the NCBI GEO Database (www.ncbi.nlm.nih.gov/projects/geo/), and HGU133A arrays from ER+, N0 and T1–T2 tumours were selected. The files were extracted and normalised using the gcrma package (Wu *et al*, 2004) for R. Three probe sets were found for SQLE (209218_at. 213562_s_at, 213577_at). The probe set 213562_s_at was omitted as probe sets carrying the suffix _s_at are designed to recognise multiple transcripts. To have a common scale for the remaining two probe sets, each value was divided by the median value of the corresponding probe set. Afterwards, the mean of the two ratios was used to classify the samples into three equally sized groups with respect to their SQLE expression. Kaplan–Meier survival curves and survival statistics were generated using the survival package for R.

## Results

**Figure 1:**
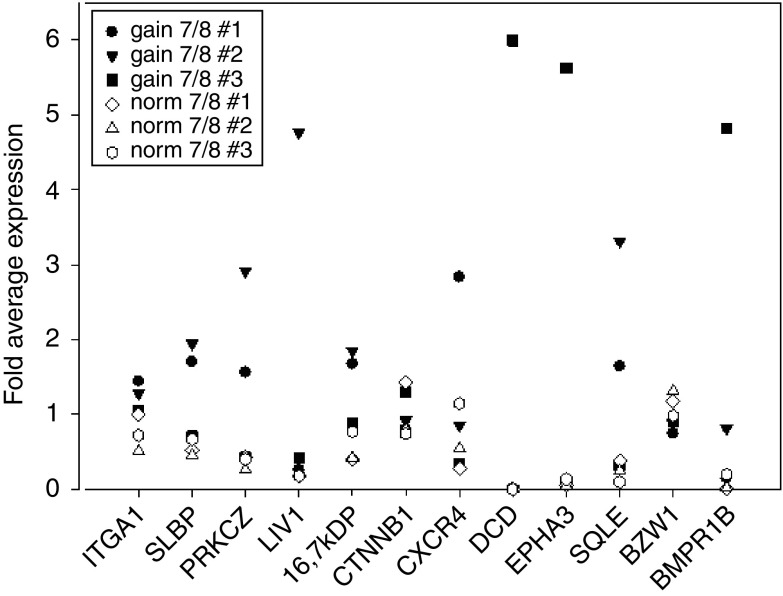
Relative expression values of the candidate gene found to be upregulated in gain 7/8 tumours by SSH as measured by real-time RT–PCR. Expression values of the single tumours were normalised to expression of 18S rRNA. For each gene product, the expression values for all six tumours were averaged and relative expression values calculated for each tumour.

The SSH-PCR approach yielded 135 mRNA species as putatively upregulated and 131 genes as putatively downregulated in gain 7/8 tumours compared to norm 7/8 tumours (Supplementary data). Of these, 60 genes were selected for validation by quantitative RT–PCR analysis by the criterion that functional and structural data were available. Sixteen gene products shown in Table 1 were confirmed to be differentially regulated in at least two of the gain 7/8 tumours compared to norm 7/8 tumours. The difference between the average expression values for gain 7/8 cases and norm 7/8 cases was more than 2.5-fold for these 16 gene products. Figure 1 shows the RT–PCR data for the 12 upregulated genes.

### Expression of SQLE, constituent of gain 7/8 tumour gene profile, predicts decreased 5-year DMFS in stage I/II breast cancer patients

To estimate the clinical relevance of these gene products associated with gain 7/8 tumours, their mRNA expression as well as that of established molecular markers (i.e. HER2, EGFR, p53 and bcl2) was quantified by real-time RT–PCR on samples from 160 patients with stage I/IIA breast cancer with known clinical records and long-term follow-up (Supplementary data). Among them, 66 developed distant metastases with a mean follow-up time of 27 months. The mean (s.d.) age of patients was 58 years (13 years), with 68.7% older than 55 years. Most tumours were of medium (41% G2) and high grades (34% G3). Fifteen per cent of the tumours were unclassified for grade.

First, we performed exploratory recursive partitioning and regression tree analysis to estimate the predictive value of expression of every gene with regard to 5-year DMFS (Zhang *et al*, 2001). Therefore, the stage I/IIA patient cohort was dichotomised for high *vs* low DMFS at more or less than 60 months, respectively. Squalene epoxidase mRNA expression levels split at the median classified 89.3% to the less than 60 months DMFS group and 78.3% to the more than 60 months DMFS group accurately. Squalene epoxidase mRNA expression levels were found to be associated with the highest predictive values of all the genes analysed.

Squalene epoxidase mRNA was detected in all 160 tumour tissues, with the tumour with the highest expression displaying a 290-fold higher level of SQLE mRNA than the lowest.

**Figure 2:**
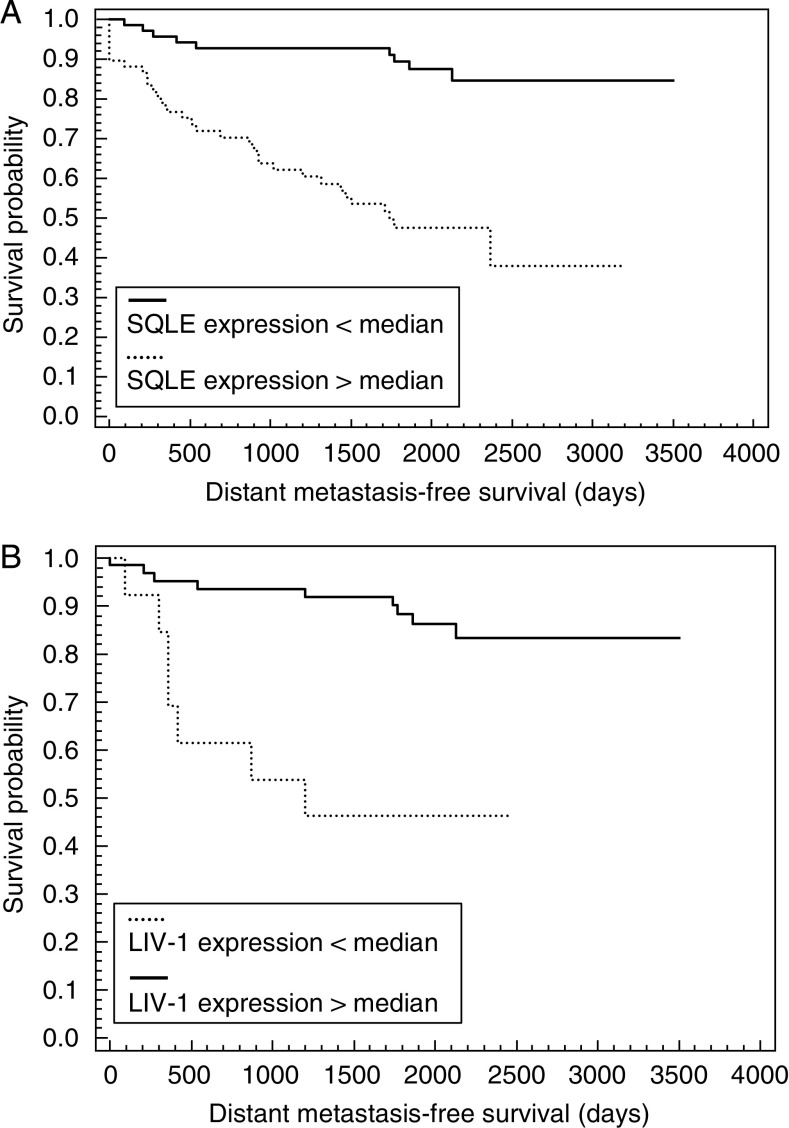
(**A**) Kaplan–Meier cumulative survival plot for distant metastasis-free survival demonstrating a significant relationship between SQLE expression and DMFS (*P*<0.0005). The median SQLE expression value dichotomised the patient group for good and poor prognosis. (**B**) Kaplan–Meier cumulative survival plot for distant metastasis-free survival demonstrating a significant relationship between LIV-1 levels and DMFS in patients with SQLE expression below median (*P*<0.0005). The median LIV-1 expression value dichotomised the patient group for good and poor prognosis.

In a second step, survival analysis according to Kaplan–Meier algorithm was performed. The survival data analysed by Kaplan–Meier test revealed a significant association between expression levels of SQLE mRNA and MFS (*P*<0.0005, Figure 2A). The 5-year DMFS rate differed by 48% between the SQLE high-expressing and SQLE low-expressing group of patients. The clinically favourable group associated with a low SQLE mRNA expression could be further divided by LIV-1 mRNA expression. In a Kaplan–Meier analysis, those tumours with high LIV-1 expression showed up with an extended time until metastatic relapse (5-year DMFS 91%), whereas the small subgroup of low LIV-1-expressing tumours (*n*=11) showed a decreased 5-year DMFS (*P*<0.0005; Figure 2B). The difference in MFS probability between both subgroups was 45%. The subgroup (*n*=30) of the poorest outcome was indicated by high SQLE and low LIV-1 expression (5-year MFS 16%). Kaplan–Meier analysis of a small subgroup of 41 patients treated with tamoxifen (5 years) and no additional radiation or chemotherapy confirmed a significant impact of SQLE mRNA expression on these tumours (*P*<0.05). The low SQLE group (*n*=30) had a 30% better estimate of DMFS than the high SQLE group (*n*=11).

### Validation of prognostic value of SQLE expression in an independent sample set

**Figure 3:**
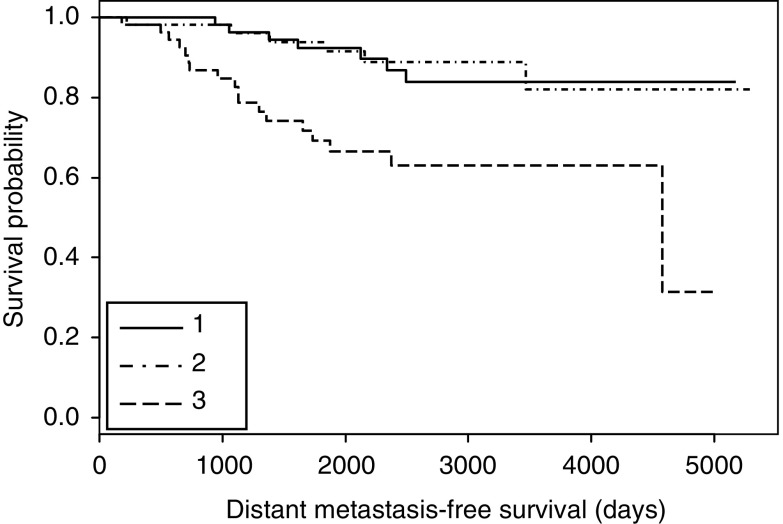
Kaplan–Meier cumulative survival plot for distant metastasis-free survival demonstrating a significant relationship between SQLE expression and DMFS (*P*<0.001). Patients were divided into three groups of equal size with patients displaying highest (3), intermediate (2) and lowest (1) expression of SQLE, respectively.

To confirm the prognostic value of SQLE mRNA, we analysed an independent validation cohort. Our findings hold true in this publicly available set of Affymetrix-based expression data (Loi *et al*, 2007). In a cohort of 162 ER+ lymph node-negative patients, tumours with the highest SQLE expression indicated a highly significantly reduced DMFS compared to the low-level expressors (*P*<0.001) (Figure 3).

### SQLE expression levels are not correlated with tumour size, grading, oestrogen receptor status and HER2 expression

Multiple *t*-testing for SQLE mRNA expression levels for comparison of the subgroups T1 *vs* T2, G2 *vs* G3, ER+ *vs* ER− and HER2+ *vs* HER2− was performed. No significant differences between the subgroups expressing SQLE mRNA above and below the median were revealed. On the other hand, LIV-1 mRNA expression level was weakly positively correlated to ER status (*P*<0.05) as already reported from other studies (Dressman *et al*, 2001), thereby confirming the validity of our sample panel.

### Significant independent influence on prognosis of the stage I and II study population for SQLE determined by multivariate Cox analysis

To evaluate the independent prognostic value of SQLE and the combination of SQLE and LIV-1, we performed multivariate survival analysis using Cox proportional hazard model as shown in Table 2. SQLE (< median *vs* > median), pT stage (pT1 *vs* pT2), histological grading (G2 *vs* G3), ER and PR status (positive *vs* negative) were entered as covariates.

Among the established clinical parameters, only tumour size (pT) reached borderline significant predictive value (*P*=0.074). The upregulation of SQLE reached high-level statistical significance (*P*=0.0014), indicating a conceivable independent impact of SQLE on stage I/II breast cancer prognosis. The risk for metastasis was increased more than five-fold for patients suffering from tumours highly expressing SQLE (Hazard ratio (HR)=5.11; *P*<0.0014). As depicted from univariate Kaplan–Meier life table analysis, the formation of a linear combined covariate from SQLE and LIV-1 mRNA expression increased HR up to 10.44 (*P*<0.0023) for the condition of SQLE mRNA expression above median and LIV-1 mRNA expression below median (SQLE(+)/LIV-1(−)). However, LIV-1 alone had no independent contribution to the metastasis risk estimate.

## Discussion

Tumours with defects on chromosome 8 have been reported in the literature as a distinct entity of breast tumours. Biological features of these tumours reflected by unfavourable clinical/pathological characteristics are likely to be driven by chromosome 8 (Rennstam *et al*, 2003). Consequently, focused gains of the chromosomal region 8q21–24 have been described as being significantly associated with alterations in disease prognosis. For instance, it has been suggested that the amplification of oncogenes on chromosome 8q (i.e., *c-MYC* and *EBAG9*) may support tumour progression. Besides 8q gains, Rennstam *et al* (2003) also report that less frequent gains of chromosome 7p were found in the 8q+ poor prognostic subgroup. We have previously described a subgroup of invasive ductal ER+ grade 3 carcinomas with chromosomal 7p gains as their cytogenetic hallmark (Korsching *et al*, 2004). These coincide with 8q gains in 50% of the cases in this unique breast cancer subgroup. However, it remains unclear whether chromosomal 7p gains represent the underlying cause of tumour progression or a mere secondary reflection thereof conceivably related to 8q gains. To gain insight into the role of these distinct cytogenetic aberrations, we compared the mRNA expression of two groups of ER+ invasive ductal breast cancers. One group consisted of tumours harbouring 7p and 8q gains and the other group was without these aberrations. The gain 7/8 tumours displayed a significantly higher number of chromosomal aberrations (mean=12) compared with the reference group (mean=5) and presented with a higher Ki67 staining as a measure of tumour cell proliferation. However, all tumours were negative for the molecular parameters HER2, EGFR and p53, which are commonly associated with an unfavourable prognosis (Fitzgibbons *et al*, 2000).

To assess the clinical value of our observations, we analysed a set of 160 randomly collected samples from stage I/IIA breast tumours with long-term follow-up and complete clinical records. Following standard guidelines, the majority of these patients would currently be treated with systemic adjuvant therapy (i.e. cytotoxic or endocrine) (Goldhirsch *et al*, 2001; National Institutes of Health Consensus Development Panel, 2001). However, a considerable number of patients will remain free of disease recurrence in the absence of additional systemic therapy. Consequently, these patients represent the subgroup of breast cancer patients most likely benefiting from improved prognosis estimation based on their genetic or genomic profile.

Among the patients in this study, 41% developed metastatic disease within a median period of follow-up of 61 months. This is higher than the assumed distant relapse rate of 30% for those tumours that could be deferred from meta-analysis of clinical trial results (Early Breast Cancer Trialists' Collaborative Group (EBCTCG), 2005). This might cause a slight overestimation of the prognostic performance of the single parameters presented in this study.

Exploratory data evaluation by means of recursive partitioning and regression tree analysis revealed SQLE to be the most relevant parameter among the candidate genes identified by SSH to dichotomise patients into high *vs* low risk of distant metastasis. Kaplan–Meier analysis of the patient cohort demonstrated a large difference in DMFS on a high significance level (*P*<0.0001). These findings were validated by the analysis of an independent patient cohort where high SQLE expression indicated low DMFS on a similar significance level (*P*<0.001).

LIV-1 expression levels might give additional information by subclassification of the SQLE prognostic groups as shown by Kaplan–Meier and Cox regression analysis. Remarkably, the prognostic value of SQLE expression even holds true for solely tamoxifen-treated patients (*P*<0.05). Considering the small size of this subgroup, it seems advisable to enroll a larger study including standardised and randomised treated patients to further validate these data.

To evaluate the independent prognostic value of SQLE and LIV-1, multivariate survival analysis using Cox proportional hazard model was applied. The upregulation of SQLE reached a high level of statistical significance (*P*=0.0014) in the multivariate model containing pT stage, histological grading, and expression of ER and PR as covariates. This strongly supports the assumption that SQLE might be an independent risk factor for metastatic relapse in stage I/II breast cancer. Importantly, HR for tumours showing both SQLE mRNA expression above and LIV-1 mRNA expression below the mean compared to others was as high as 10.44. This may serve as a potential rationale for a clinical trial setting in which patients would be stratified to receive endocrine therapy with treatment with tamoxifen or aromatase inhibitors based on SQLE and LIV-1 expression. LIV-1 has been demonstrated to be correlated positively with ER expression and negatively with histological grade (Taylor *et al*, 2007). Corroborating the data presented here, LIV-1 has also been demonstrated to predict longer relapse-free survival and OS in ER+ breast cancer (Kasper *et al*, 2005). The same study indicated that the predictive value of LIV-1 is dependent on other prognostic markers. Our data identified SQLE to be one of these factors, even though the functional relationship remains unclear.

Studies indicated that LIV-1 plays a role in tissue differentiation and epithelial mesenchymal transition (Taylor *et al*, 2004; Zhao *et al*, 2007). This biological role is in compliance with the negative correlation of LIV-1 with histological grade. Histological grade represents the histomorphological correlate of (breast) cancer differentiation and predicts an unfavourable prognosis (Fukutomi and Akashi-Tanaka, 2002; Liedtke *et al*, 2008).

Published data on SQLE suggest a biochemical rationale for a protective effect in SQLE-negative tumours as observed in this study and the prognostic and predictive implications. Distribution studies in human tissues show a very low expression of SQLE in non-cholesterogenic tissues (including the mammary gland) compared with cholesterogenic tissues like the liver. It is assumed that the reaction catalysed by SQLE is the rate limiting step in cholesterol biosynthesis, in post-HMG CoA regulation (Astruc *et al*, 1977; Tabacik *et al*, 1981). This is supported by the fact that cell growth inhibited by a specific SQLE inhibitor (NB-598) could be rescued by small amounts of cholesterol added to the culture medium (Xu *et al*, 2005).

Given that SQLE resides on chromosome 8q24.1 (Nagai *et al*, 1997), which is commonly involved in 8q gains in breast cancer, it seems conceivable that tumour-promoting or -initiating effects that 8q gains may have involve deregulation of SQLE expression. Chin *et al* (2007) demonstrated a strong statistical correlation between chromosomal 8q gains and upregulation of SQLE expression in human breast cancer, suggesting a direct relation between gene copy numbers and expression. Even though the connecting link between SQLE expression and cytogenetic instability remains unclear, it might be speculated that an increase in proliferation activity induced by a loop of trace amounts of cholesterol that is self-sufficient would also increase the likelihood for genetic aberrations.

We identified mRNA expression of SQLE, located on chromosome 8q24.1, to be associated with high-risk ER+ breast cancer cases. Squalene epoxidase mRNA expression was able to define a patient subgroup at significantly increased risk of early onset of metastasis among ER+ stage I/II breast cancer. Furthermore, SQLE expression remained a significant prognostic factor for increased/decreased DMFS, independent of established prognostic factors such as tumour size and grade.

The findings presented here might be used in the future to identify patients with ER+ breast cancer, which would benefit from additional treatment besides encdocrine therapy.

## Figures and Tables

**Table 1:** List of validated genes found to be differentially regulated in gain 7/8 tumours compared to norm 7/8 tumours

	**Annotation of cDNA**	**Accession no.**	**Locus**
*Genes upregulated in gain 7/8 tumours*			
1	CXCR4: chemokine (C-X-C motif) receptor 4	NM_003467	2q21
2	EPHA3: EphA3	NM_005233	3p11.2
3	CTNNB1: catenin (cadherin-associated protein) beta 1, 88 kDa	NM_001904	3p21
4	SLBP: stem-loop (histone)-binding protein	NM_006527	4p16.3
5	BMPR1B: bone morphogenetic protein receptor, type IB	NM_001203	4q22–q24
6	ITGA1: integrin, alpha 1	NM_181501	5p11
7	CHCHD2; 16.7 kDa protein (LOC51142)	NM_016139	7p14.1
8	BZW1: basic leucine zipper and W2 domains 1	NM_014670	8q22.2–q23/ 2q33
9	PRKCZ (14-3-3 zeta)	NM_145690	8q23.1
10	SQLE: squalene epoxidase	NM_003129	8q24.1
11	DCD: dermcidin	NM_053283	12q13
12	LIV-1: LIV-1 protein, oestrogen-regulated	NM_012319	18q12.1
			
*Genes downregulated in gain 7/8 tumours*			
13	CALM2: calmodulin 2 (phosphorylase kinase, delta)	NM_001743	2p21
14	GLI3: GLI-Kruppel family member GLI3	NM_000168	7p13
15	CXCL12/cytokine SDF-1-beta	NM_000609	10q11.1
16	BUB3: budding uninhibited by benzimidazoles 3 homologue	NM_004725	10q26

**Table 2:** Multivariate analysis for DMFS in a validation set of 160 stage I/II breast cancer patients

	**Univariate (*n*=160)**		**Multivariate (*n*=160)**	
**Covariate**	**HR (95% CI)**	***P*-value**	**HR (95% CI)**	***P*-value**
pT1 *vs* pT2	2.18 (0.93–5.1)	0.074	2.81 (1.09–7.25)	0.033
G2 *vs* G3	NA	NA	0.53 (0.20–1.41)	0.20
ER+ *vs* ER−	0.75 (0.32–1.76)	0.52	1.90 (0.60–6.06)	0.28
SQLE	5.11 (1.88–13.9)	0.0014	5.72 (2.20–16.2)	0.001
LIV-1	0.49 (0.17–1.46)	0.2	0.38 (0.11–1.32)	0.13
SQLE(+)/LIV-1(+)	5.27 (1.73–16.1)	0.0035	NA	NA
SQLE(+)/LIV-1(−)	10.44 (2.31–47.2)	0.0023	NA	NA

CI=confidence interval; DMFS=distant metastasis-free survival; HR=hazards ratio; SQLE=squalene epoxidase; NA=Not applicable.
