# Supplementary material for: Squalene epoxidase, located on chromosome 8q24.1, is upregulated in 8q+ breast cancer and indicates poor clinical outcome in stage I and II disease
Source: Br J Cancer. 2008 Aug 12;99(5):774–80. doi: 10.1038/sj.bjc.6604556 (PMC2528137; doi:10.1038/sj.bjc.6604556)
Supplement: Supplementary data [file 6604556x1.doc]

## Supplemental data

## SI: Lists of gene products found to be differentially expressed in 7p+/8q+ tumors compared to 7p/8q normal tumors.

Table S1: List of 135 genes found to be upregulated in 7p+/8q+ tumors compared to 7p/8q normal tumors.

| **Genes upregulated in 7p+/8q+** | | | |
| --- | --- | --- | --- |
|  | **cDNA** | **Accession#** | **Locus** |
| 1 | VAV3: vav 3 oncogene (splicing variant) | NM_006113 | 1p13.1 |
| 2 | Similar to small inducible cytokine B subfamily (Cys-X-Cys motif), member 13 (B-cell chemoattractant | BC012589 | 1p31.1 |
| 3 | CD53: transmembrane glycoprotein | L11671S2 | 1p31-p12 |
| 4 | SMARCF1: SWI/SNF related, matrix associated, actin dependent regulator of chromatin, subfamily f, member 1 | NM_006015 | 1p35.2 |
| 5 | HMGN2: high-mobility group nucleosomal binding domain 2 | NM_005517 | 1p36.1 |
| 6 | EIF4G3: eukaryotic translation initiation factor 4 gamma, 3 | NM_003760 | 1p36.12 |
| 7 | NICE-3 protein | NM_015449 | 1q21.2 |
| 8 | YAP: YY1 associated protein | NM_139120 | 1q21.2 |
| 9 | AOP2: anti-oxidant protein 2 (non-selenium glutathione peroxidase, acidic calcium-independent phospholipase A2) | NM_004905 | 1q23.3 |
| 10 | H3F3A: H3 histone, family 3A | M11353 | 1q41 |
| 11 | SRP9 : signal recognition particle 9kDa | NM_003133 | 1q41 |
| 15 | similar to ribonucleoprotein | XM_165561 | 2 |
| 16 | hypothetical protein LOC220988 | XM_165561 | 2 |
| 17 | CCT7: chaperonin containing TCP1, subunit 7 (eta) | NM_006429 | 2p12 |
| 18 | likely ortholog of mouse metastasis associated 3 | BC004227 | 2p21 |
| 19 | SLC4A1AP: solute carrier family 4 (anion exchanger), member 1, adaptor protein | NM_018158 | 2p23.3 |
| 20 | MRPL19: mitochondrial ribosomal protein L19 | NM_014763 | 2q11.1-q11.2 |
| 21 | CXCR4: chemokine (C-X-C motif) receptor 4 | NM_003467 | 2q21 |
| 22 | COL3A1 : collagen, type III, alpha 1 (Ehlers-Danlos syndrome type IV, autosomal dominant) | NM_000090 | 2q31 |
| 23 | HSPC133 protein | NM_014168 | 2q31.1 |
| 24 | NEB: nebulin | NM_004543 | 2q31-q32 |
| 25 | TMEFF2: transmembrane protein with EGF-like and two follistatin-like domains 2 | AB004064 | 2q32.3 |
| 26 | cDNA: FLJ21652 fis, | AK025305 | 3 |
| 27 | EPHA3: EphA3 | NM_005233 | 3p11.2 |
| 28 | FLNB: filamin B, beta (actin binding protein 278) | NM_001457 | 3p14.3 |
| 29 | CTNNB1: catenin (cadherin-associated protein), beta 1, 88kDa | NM_001904 | 3p21 |
| 30 | UQCRC1: ubiquinol-cytochrome c reductase core protein I | NM_003365 | 3p21.3 |
| 31 | DDX30: DEAD/H (Asp-Glu-Ala-Asp/His) box polypeptide 30 | NM_138615 | 3p21.31 |
| 32 | LSM3: LSM3 homolog, U6 small nuclear RNA associated (S. cerevisiae) | NM_014463 | 3p25.1 |
| 33 | PCNP: PEST-containing nuclear protein | NM_020357 | 3q13.11 |
| 34 | CD10: membrane metallo-endopeptidase | NM_007287 | 3q25.1-q25.2 |
| 35 | IGJ: immunoglobulin J polypeptide, linker protein for immunoglobulin alpha and mu polypeptides | NM_144646 | 4 |
| 36 | cDNA DKFZp564A072 | AL049969 | 4 |
| 37 | cDNA FLJ23044 fis | AK026697 | 4 |
| 38 | SYNPO2: synaptopodin 2 | XM_050219 | 4 |
| 39 | cDNA FLJ31439 fis | AK056001 | 4 |
| 40 | SLBP: stem-loop (histone) binding protein | NM_006527 | 4p16.3 |
| 41 | CXCL13: chemokine (C-X-C motif) ligand 13 (B-cell chemoattractant) | NM_006419 | 4q21 |
| 42 | Similar to small inducible cytokine B subfamily (Cys-X-Cys motif), member 13 (B-cell chemoattractant) | AF044197 | 4q21 |
| 43 | HNRPD: heterogeneous nuclear ribonucleoprotein D | NM_031369 | 4q21.1-q21.2 |
| 44 | BMPR1B: bone morphogenetic protein receptor, type IB | NM_001203 | 4q22-q24 |
| 45 | H2AFZ: H2A histone family, member Z | NM_002106 | 4q24 |
| 46 | NFKB1: nuclear factor of kappa light polypeptide gene enhancer in B-cells 1 (p105) | NM_003998 | 4q24 |
| 47 | CCNA2: cyclin A2 | NM_001237 | 4q25-q31 |
| 48 | HMGB2: high-mobility group box 2 | NM_002129 | 4q31 |
| 49 | KIAA0882 protein | XM_093895 | 4q31.1 |
| 50 | SH3 domain protein D19 | XM_098238 | 4q31.23 |
| 51 | ITGA1: integrin, alpha 1 | NM_181501 | 5p11 |
| 52 | transcription elongation factor B polypeptide 1-like | NM_003197 | 5q31 |
| 53 | DUSP1: dual specificity phosphatase 1 | NM_004417 | 5q34 |
| 54 | HSPCB: heat shock 90kDa protein 1, beta | NM_007355 | 6p12 |
| 55 | SLC29A1: solute carrier family 29 (nucleoside transporters), member 1 | NM_004955 | 6p21.1-p21.2 |
| 56 | PHIP: pleckstrin homology domain interacting protein | NM_017934 | 6q14 |
| 57 | ZNF292: zinc finger protein 292 | XM_048070 | 6q16.1 |
| 58 | CD164, Sialomucin | NM_006016 | 6q21 |
| 59 | MLLT4: myeloid/lymphoid or mixed-lineage leukemia (trithorax homolog, Drosophila); translocated to 4 | NM_005936 | 6q27 |
| 60 | 16.7Kd protein (LOC51142) | NM_016139 | 7p14.1 |
| 61 | PEG10: paternally expressed 10 | NM_015068 | 7q21 |
| 62 | TRIP6: thyroid hormone receptor interactor 6, EPHB4: EphB4, ZAN: zonadhesin | AF312032 | 7q22 |
| 63 | TES: testis derived transcript (3 LIM domains) | NM_015641 | 7q31.2 |
| 64 | cDNA FLJ10943 fis | AK001805 | 8 |
| 65 | KIAA0196 | NM_014846 | 8p22 |
| 66 | cDNA DKFZp667D0 | AL512749 | 8p22-p21.3 |
| 67 | TCEB1: transcription elongation factor B (SIII), polypeptide 1 (15kDa, elongin C) | AK057889 | 8q13.3 |
| 68 | SGKL: serum/glucocorticoid regulated kinase-like | NM_013257 | 8q12.3-8q13.1 |
| 69 | KIAA1077 | AB029000 | 8q13.1 |
| 70 | BZW1: basic leucine zipper and W2 domains 1 | NM_014670 | 8q22.2-q23/ 2q33 |
| 71 | FZD6: frizzled homolog 6 (Drosophila) | NM_003506 | 8q22.3-q23.1 |
| 72 | SDC2: syndecan 2 (heparan sulfate proteoglycan 1, cell surface-associated, fibroglycan) | NM_002998 | 8q22-q23 |
| 73 | EIF3S6: eukaryotic translation initiation factor 3, subunit 6 48kDa | NM_001568 | 8q22-q23 |
| 74 | 14-3-3 zeta | NM_145690 | 8q23.1 |
| 75 | TRPS1: trichorhinophalangeal syndrome I | NM_014112 | 8q24.1 |
| 76 | SQLE: squalene epoxidase | NM_003129 | 8q24.1 |
| 77 | SET: SET translocation (myeloid leukemia-associated) | NM_003011 | 9q34 |
| 78 | FLJ22071 fis | AK025724 | 10 |
| 79 | ALOX5: arachidonate 5-lipoxygenase | NM_000698 | 10q11.2 |
| 80 | HNRPF: heterogeneous nuclear ribonucleoprotein F | NM_004966 | 10q11.21-q11.21 |
| 81 | FER1L3: fer-1-like 3, myoferlin (C. elegans) | NM_013451 | 10q24 |
| 82 | KNSL1: kinesin-like 1 | NM_004523 | 10q24.1 |
| 83 | RGS10: regulator of G-protein signalling 10 | NM_002925 | 10q25 |
| 84 | CSTF3: cleavage stimulation factor, 3' pre-RNA, subunit 3, 77kDa | NM_001326 | 11p13 |
| 85 | heat shock 90kDa protein 1, alpha | AF203815 | 11q13 |
| 86 | hypothetical protein MGC2477 | NM_024099 | 11q13.1 |
| 87 | MTL5: metallothionein-like 5, testis-specific (tesmin) | NM_004923 | 11q13.2-q13.3 |
| 88 | KIAA0102 gene product | NM_014752 | 11q13.3 |
| 89 | KIAA0102 gene product | NM_014752 | 11q13.3 |
| 90 | hypothetical protein MGC13040 | NM_032930 | 11q22.1 |
| 91 | SLN: sarcolipin | NM_003063 | 11q22-q23 |
| 92 | DLAT: dihydrolipoamide S-acetyltransferase (E2 component of pyruvate dehydrogenase complex) | NM_001931 | 11q23.1 |
| 93 | KIAA1025 protein | AB028948 | 12 |
| 94 | CSDA: cold shock domain protein A | NM_003651 | 12p13.1 |
| 95 | C3AR1: complement component 3a receptor 1 | NM_004054 | 12p13.32 |
| 96 | ATP synthase, H+ transporting, mitochondrial F1 complex, beta polypeptide (ATP5B) | NM_001686 | 12p13-qter |
| 97 | MYL6: myosin, light polypeptide 6, alkali, smooth muscle and non-muscle | NM_079423 | 12q12 |
| 98 | TEGT: testis enhanced gene transcript (BAX inhibitor 1) | NM_003217 | 12q12-q13 |
| 99 | DCD: Dermcidin | NM_053283 | 12q13 |
| 100 | SLC11A2: solute carrier family 11 (proton-coupled divalent metal ion transporters), member 2 | NM_000617 | 12q13 |
| 101 | KIAA1463 protein | AB040896 | 12q13.13 |
| 102 | MYBPC1: myosin binding protein C, slow type | NM_002465 | 12q23.2 |
| 103 | RPLP0: ribosomal protein, large, P0 | NM_001002 | 12q24.2 |
| 104 | tumor rejection antigen (gp96) 1 (TRA1) | NM_003299 | 12q24.2-q24.3 |
| 105 | TG737: Probe hTg737 (polycystic kidney disease, autosomal recessive) | NM_006531 | 13q12.1 |
| 106 | hypothetical protein FLJ10154 | NM_018011 | 13q33.3 |
| 107 | KIAA0391 gene product | NM_014672 | 14q13.1 |
| 108 | STRN3: striatin, calmodulin binding protein 3 | NM_014574 | 14q13-q21 |
| 109 | ZFYVE1: zinc finger, FYVE domain containing 1 | NM_178441 | 14q22-q24 |
| 110 | FNTB: farnesyltransferase, CAAX box, beta | NM_002028 | 14q23-q24 |
| 111 | SEL1L: sel-1 suppressor of lin-12-like (C. elegans) | NM_005065 | 14q24.3-q31 |
| 112 | similar to TSG118.1 protein | XM_058778 | 16p12.3 |
| 113 | ADP-ribosylation factor-like 6 interacting protein | D31885 | 16p12-p11.2 |
| 114 | SRRM2: serine/arginine repetitive matrix 2 | AF201422 | 16p13.3 |
| 115 | CDA08: T-cell immunomodulatory protein | NM_030790 | 16q11.2-q12.1 |
| 116 | NIP30: NEFA-interacting nuclear protein NIP30 | AF356585 | 16q13 |
| 117 | KIAA1554 protein | XM_170834 | 17 |
| 118 | ALDH3A2: aldehyde dehydrogenase 3 family, member A2 | NM_000382 | 17p11.2 |
| 119 | COL1A1: collagen, type I, alpha 1 | NM_000088 | 17q21.3-q22.1 |
| 120 | P15RS: hypothetical protein FLJ10656 | AF419845 | 18 |
| 121 | TUBB-5: tubulin beta-5 | NM_032525 | 18p |
| 122 | HCCA3: hepatocellular carcinoma susceptibility protein | AF276707 | 18p11.21 |
| 123 | USP14: ubiquitin specific protease 14 (tRNA-guanine transglycosylase) | NM_005151 | 18p11.32 |
| 124 | LIV-1: LIV-1 protein, estrogen regulated | NM_012319 | 18q12.1 |
| 125 | MADH7: MAD, mothers against decapentaplegic homolog 7 (Drosophila) | NM_005904 | 18q12.3 |
| 126 | hypothetical protein FLJ14640 | NM_032816 | 19q13.12 |
| 127 | RPL13A: ribosomal protein L13a | NM_012423 | 19q13.3 |
| 128 | cDNA FLJ90795 fis | AK075276 | 19q13.42 |
| 12 | EPB41L1: erythrocyte membrane protein band 4.1-like 1 | NM_012156 | 20q11.2-q12 |
| 13 | ATP5E: ATP synthase, H+ transporting, mitochondrial F1 complex, epsilon subunit | NM_006886 | 20q13.3 |
| 14 | PRAME: preferentially expressed antigen in melanoma | NM_006115 | 22q11.22 |
| 129 | TUBB2: tubulin, beta, 2 | NM_006088 | X |
| 130 | RBBP7: retinoblastoma binding protein 7 | NM_002893 | Xp22.13 |
| 131 | SH3BGRL: SH3 domain binding glutamic acid-rich protein like | NM_003022 | Xq13.3 |
| 132 | COL4A5: collagen, type IV, alpha 5 (Alport syndrome) | NM_033380 | Xq22 |
| 133 | NRF: NF-kappa B-repressing factor | NM_017544 | Xq25 |
| 134 | Similar to cytochrome c oxidase III | BC013932 | mitochondrial |
| 135 | Cytochrom B | J01415 | mitochondrial |

Table S2: List of 131 genes found to be downregulated in 7p+/8q+ tumors compared to 7p/8q normal tumors.

| **Genes downregulated in 7p+/8q+** | | | |
| --- | --- | --- | --- |
|  | **cDNA** | **Accession#** | **Locus** |
| 1 | hypothetical protein FLJ32001 | NM_152609 | 1 |
| 2 | neuroblastoma RAS viral (v-ras) oncogene homolog | NM_007158 | 1p22 / 1p13.2 |
| 3 | NFIA: Homo sapiens nuclear factor I/A | XM_046827 | 1p31.3-31.2 |
| 4 | ribosomal protein S8 | NM_001012 | 1p34.1-p32 |
| 5 | RIZ1: zinc-finger DNA-binding protein | NM_012231 | 1p36 |
| 6 | peroxisomal farnesylated protein | NM_002857 | 1q22 |
| 7 | ATP1B: Na,K-ATPase beta subunit | U16799 | 1q22-q25 |
| 8 | C1orf28: chromosome 1 open reading frame 28 | NM_024529 | 1q25 |
| 9 | SRP9: signal recognition particle 9kD | NM_003133 | 1q32.2 |
| 10 | similar to putative (LOC116228) | XM_057659 | 1q44 |
| 11 | rearranged Ig kappa light chain variable region (I.26) | X72443 | 2 |
| 12 | cDNA FLJ14439 fis | AK027345 | 2 |
| 13 | hypothetical protein FLJ10379 | NM_018079 | 2p21 |
| 14 | CALM2: calmodulin 2 (phosphorylase kinase, delta) | NM_001743 | 2p21 |
| 15 | LOC151103 (LOC151103) | XM_098004 | 2p22.1 |
| 16 | CBF: CCAAT-box-binding factor | M37197 | 2p22.3 |
| 17 | D2LIC: dynein 2 light intermediate chain | XM_031519 | 2p25.1-p24.1 |
| 18 | MGC10993: hypothetical protein MGC10993 | NM_030577 | 2q21.2 |
| 19 | COL3A1: collagen, type III, alpha 1 (Ehlers-Danlos syndrome type IV, autosomal dominant) | NM_000090 | 2q31 |
| 20 | COL3A1: collagen, type III, alpha 1 | XM_057363 | 2q31 |
| 21 | FN1: fibronectin 1 | NM_054034 | 2q34 |
| 22 | ARHA: ras homolog gene family, member A | NM_001664 | 3p21.3 |
| 23 | CKLFSF8: chemokine-like factor super family 8 | NM_178868 | 3p22.2 |
| 24 | NBEA: neurobeachin | NM_015678 | 3q13 |
| 25 | NDUFB4: NADH dehydrogenase (ubiquinone) 1 beta subcomplex 4 | NM_004547 | 3q13.33 |
| 26 | AGTR1: angiotensin II receptor, type 1 | NM_009585 | 3q21-q25 |
| 27 | RBP1: retinol binding protein 1, cellular | NM_002899 | 3q23 |
| 28 | RPL35A: ribosomal protein L35a | NM_000996 | 3q29-qter |
| 29 | OCIA: ovarian carcinoma immunoreactive antigen | NM_017830 | 4p12-cen |
| 30 | CENTD1: centaurin, delta 1 | NM_015230 | 4p15.1 |
| 31 | UGT2B11: UDP glycosyltransferase 2 family, polypeptide B11 | NM_001073 | 4q13.1 |
| 32 | IMMUNOGLOBULIN J CHAIN | XM_059628 | 4q13.3 |
| 33 | PDGFC: platelet derived growth factor C (Fallotein) | NM_016205 | 4q21 |
| 34 | SCYB10: small inducible cytokine subfamily B (Cys-X-Cys), member 10 | NM_001565 | 4q21 |
| 35 | EIF4E: eukaryotic translation initiation factor 4E | NM_001968 | 4q21-q25 |
| 36 | SLC39A8: solute carrier family 39 (zinc transporter), member 8, up-regulated by BCG-CWS | BC012125 | 4q22-q24 |
| 37 | ANXA5: annexin A5 (placental anticoagulant protein I) | NM_001154 | 4q28-q32 |
| 38 | NPY1R: neuropeptide Y receptor Y1 | NM_000909 | 4q31.3-q32 |
| 39 | GLRA3: glycine receptor, alpha 3 | NM_006529 | 4q33-q34 |
| 40 | GPM6A: glycoprotein M6A | NM_005277 | 4q34 |
| 41 | cDNA DKFZp762O1615 | AL359558 | 5 |
| 42 | AIPC: PDZ domain-containing protein AIPC | AF338650 | 5p14.1 |
| 43 | MRPS30: mitochondrial ribosomal protein S30 | NM_016640 | 5q11 |
| 44 | BTF2: basic transcription factor 2, p44 subunit | NM_001515 | 5q12.2-q13.3 |
| 45 | SLC12A2: solute carrier family 12 (sodium/potassium/chloride transporters), member 2 | NM_001046 | 5q23.3 |
| 46 | HTGN29: HTGN29 protein | NM_020199 | 5q31.1 |
| 47 | EGR1: early growth response 1 | NM_001964 | 5q31.1 |
| 48 | MHC lymphocyte antigen (DRB1*1302) | HUMMH1302D | 6p21.3 |
| 49 | DDAH2: dimethylarginine dimethylaminohydrolase 2 | NM_013974 | 6p21.3 |
| 50 | HLA-DR beta-chain | M15178 | 6p21.3 |
| 51 | BF: B-factor, properdin | NM_001710 | 6p21.3 |
| 52 | MYLIP: myosin regulatory light chain interacting protein | NM_013262 | 6p23-p22.3 |
| 53 | COL12A1: collagen, type XII, alpha 1 | NM_080645 | 6q12-q13 |
| 54 | EEF1A1: eukaryotic translation elongation factor 1, alpha 1 | NM_001402 | 6q14.1 |
| 55 | MTO1 homolog: mitochondrial translation optimization 1 homolog | XM_087899 | 6q14.1 |
| 56 | CD73: 5' nucleotidase | NM_002526 | 6q14-q21 |
| 57 | AMD1: S-adenosylmethionine decarboxylase 1 | NM_001634 | 6q21-q22 |
| 58 | SGK: serum/glucocorticoid regulated kinase | BC001263 | 6q23 |
| 59 | TAB2: TAK1-binding protein 2 | NM_145342 | 6q25.1-q25.3 |
| 60 | GLI3: GLI-Kruppel family member GLI3 | NM_000168 | 7p13 |
| 61 | RAC1: ras-related C3 botulinum toxin substrate 1 (rho family, small GTP binding protein Rac1) | AJ132695 | 7p22/4/2 |
| 62 | hypothetical protein PRO1722 | BC005981 | 7q |
| 63 | COL1A2: collagen, type I, alpha 2 | XM_029245 | 7q22.1 |
| 64 | DNAJB9: DnaJ (Hsp40) homolog, subfamily B, member 9 | NM_012328 | 7q31/14q24.2-q24.3 |
| 65 | NADH dehydrogenase (ubiquinone) 1 alpha subcomplex, 5, 13kDa (NDUFA5), mitochondrial | NM_005000 | 7q32 |
| 66 | STC1: stanniocalcin 1 | NM_003155 | 8p21-p11.2 |
| 67 | FBXO25: F-box only protein 25 (unknown transcript) | NM_012173 | 8p23.3 |
| 68 | RPL7: ribosomal protein L7 | NM_000971 | 8q13.2 |
| 69 | RIPK2: receptor-interacting serine-threonine kinase 2 | AF027706 | 8q21 |
| 70 | CTHRC1: collagen triple helix repeat containing 1 | NM_138455 | 8q22.3 |
| 71 | COBW-like protein (LOC55871) aka dopamine-responsive protein | XM_036311 | 9p22.2 |
| 72 | ASPN: asporin (LRR class 1) | NM_017680 | 9q22 |
| 73 | GSN: gelsolin | NM_000177 | 9q33 |
| 74 | ATP6V1G1: ATPase, H+ transporting, lysosomal 13kDa, V1 subunit G isoform 1 | NM_004888 | 9q33.1 |
| 75 | bromodomain containing 3 | AC002323 | 9q34 |
| 76 | CXCL12/cytokine SDF-1-beta | NM_000609 | 10q11.1 |
| 77 | Sec23-interacting protein p125 | AK001135 | 10q25-q26 |
| 78 | BUB3: budding uninhibited by benzimidazoles 3 homolog (yeast) | NM_004725 | 10q26 |
| 79 | HSP90alpha: heat shock 90kDa protein 1, alpha | D87666 | 11cen-q12.3 / 14q32.33 |
| 80 | E74-like factor 5 | AF115403 | 11p14 |
| 81 | cDNA PSEC0048 fis | AK075362 | 11q |
| 82 | DKFZP564O0423 protein | XM_166254 | 11q13.4 |
| 83 | NADH dehydrogenase (ubiquinone) 1 | NM_004549 | 11q13.5 |
| 84 | CS box-containing WD protein | NM_018639 | 12 |
| 85 | PTHLH: parathyroid hormone-like hormone | NM_002820 | 12p12.1-p11.2 |
| 86 | C1S: complement component 1, s subcomponent | NM_001734 | 12p13 |
| 87 | PTPRO/GLEPP1: protein tyrosine phosphatase, receptor type, O/ glomerular epithelial protein 1 | NM_030671 | 12p13.3-p13.2 /12p13-p12 |
| 88 | contactin 1/2 | NM_001843/ | 12q11-q12 |
| 89 | cDNA FLJ36720 fis | AK094039 | 12q12 |
| 90 | EPLIN/SREBP3: epithelial protein lost in NEO plasm beta aka sterol regulatory element binding protein 3 | NM_016357 | 12q13 |
| 91 | decorin | NM_001920 | 12q13.2 |
| 92 | KIAA0546 protein | XM_049055 | 12q15 |
| 93 | DSPG3: dermatan sulfate proteoglycan 3 | XM_012240 | 12q21 |
| 94 | VEZATIN: transmembrane protein vezatin | NM_017599 | 12q21.33 |
| 95 | general transcription factor IIIA | U20272 | 13q12.3-q13.1 |
| 96 | translationally controlled tumor protein | X16064 | 13q12-q14 |
| 97 | LIG4: ligase IV, DNA, ATP-dependent | NM_002312 | 13q33-q34 |
| 98 | ING1: inhibitor of growth family, member 1 | NM_005537 | 13q34 |
| 99 | cDNA FLJ11685 fis |  | 14 |
| 100 | SEC10L1: SEC10-like 1 (S. cerevisiae) | NM_006544 | 14q22.2 |
| 101 | ERF-1: zinc finger protein 36, C3H type-like 1 (ZFP36L1) aka BRF1, cMG1, Berg36, TIS11B | NM_004926 | 14q22-q24 |
| 102 | RASGRP1: RAS guanyl releasing protein 1 (calcium and DAG-regulated) | NM_005739 | 15q15 |
| 103 | SORD: sorbitol dehydrogenase | XM_058257 | 15q15.3 |
| 104 | B2M: beta-2-microglobulin | NM_004048 | 15q21-q22.2 |
| 105 | CLIP: cartilage intermediate layer protein, nucleotide pyrophosphohydrolase | NM_003613 | 15q22 |
| 106 | RNA polymerase I transcription factor aka LAT1-3TM protein / carbonic anhydrase VA Pseudogene/mitochondrial | AC003007 AC009086 | 16p12/ 16q24.3 |
| 107 | TNFRSF17: tumor necrosis factor receptor superfamily 17 | NM_001192 | 16p13.1 |
| 108 | GABARAPL2: GABA(A) receptor-associated protein-like 2 | BC005985 | 16q22.3-q24.1 |
| 109 | UBB: ubiquitin B | NM_018955 | 17p12-p11.2 |
| 110 | hypothetical protein MGC14376 (MGC14376), | NM_032895 | 17p13.3 |
| 111 | putative ATP dependent RNA helicase | AF319521 | 17q23.2 |
| 112 | nuk_34 mRNA for translation initiation factor/mitochondriales Material: Chimäre | X79538/ | 17q25.3/mitochondrial |
| 113 | CYP2A3: Cytochrom P-450 IIA3 | NM_000762 | 19q13.2 |
| 114 | HSPC051: ubiquinol-cytochrome c reductase complex (7.2 kD) | NM_013387 | 19q13.2 - 22cen-q12.3 |
| 115 | ribosomal protein S9 mRNA | U14971 | 19q13.4 |
| 116 | NAT5/S106 CALCYCLIN: N-acetyltransferase 5 (ARD1 homolog, S. cerevisiae) | NM_016100 | 20p11.22 |
| 117 | TOP1: topoisomerase (DNA) I | NM_003286 | 20q12-q13.1 |
| 118 | stress 70 protein chaperone, microsome-associated, 60kD, ATPase | NM_006948 | 21q11 |
| 119 | ATP synthase, H+ transporting, mitochondrial F0 complex, subunit F6 | NM_001685 | 21q21.1 |
| 120 | cDNA FLJ10798 fis/ HYPOTHETICAL 12.7 KD HISTONE H2A RELATED PROTEIN | AK001660 | 21q22.13 |
| 121 | HMGN1: high-mobility group nucleosome binding domain 1 | NM_004965 | 21q22.2 / 14q24.3 |
| 122 | SMT3H1: SMT3 suppressor of mif two 3 homolog 1 (yeast) | NM_006936 | 21q22.3 |
| 123 | immunoglobulin lambda light chain mRNA | BC022823 | 22 |
| 124 | XBP1: X-box binding protein 1 | NM_005080 | 22q12.1 |
| 125 | ST13/UBXD2: suppression of tumorigenicity 13 (colon carcinoma)(Hsp70 interacting protein) /UBX domain-containing 2 | NM_003932/ XM_043196 | 22q13.2/ 2q21.2 |
| 126 | TMSB4: thymosin, beta 4 | BC022857 | X |
| 127 | RAD54 homolog: alpha thalassemia/mental retardation syndrome X-linked (S. cerevisiae), Zinc finger helicase | NM_000489 | Xq13.1-q21.1 |
| 128 | HNRPH2: heterogeneous nuclear ribonucleoprotein H2 (H') | NM_019597 | Xq22 |
| 129 | TCEAL1/p21: transcription elongation factor A (SII)-like 1 | XM_010124 | Xq22.1 |
| 130 | RPL7A: ribosomal protein L7a | NM_000972 |  |
| 131 | cDNA DKFZp666G145 | AL833729 |  |

**SII: Clinical information of patients included in real time RT-PCR expression profiling.**

## Table S3: List of 161 patients providing relevant clinical information including treatments. Abbreviations used: CMF: Cyclophosphamide, methotrexate and fluorouracil combination, Tam: Tamoxifen, rad: radiation, EC: Epirubicin and cyclophosphamid combination, FEC: Fluorouracil (5FU), epirubicin and cyclophosphamide combination, GNRH: Gonadotropin Releasing Hormone

| **Patient #** | **Age** | **T** | **N** | **M** | **Grade** | **ER status** | **PgR status** | **Therapy** |
| --- | --- | --- | --- | --- | --- | --- | --- | --- |
| 1 | 36 | 2 | 0 | 0 | 3 | - | - | 0 |
| 2 | 40 | 1 | 0 | 0 | 2 | - | - | 0 |
| 3 | 46 | 1 | 0 | 0 | 2 | - | - | 0 |
| 4 | 50 | 1 | 0 | 0 | 1 | + | - | 0 |
| 5 | 49 | 2 | 0 | 0 | 2 | + | + | 0 |
| 6 | 49 | 1 | 0 | 0 | 2 | + | + | 0 |
| 7 | 41 | 2 | 0 | 0 | 0 | + | + | 0 |
| 8 | 55 | 1 | 0 | 0 | 0 | + | + | 0 |
| 9 | 34 | 1 | 0 | 0 | 2 | + | + | 0 |
| 10 | 48 | 2 | 0 | 0 | 3 | - | - | 0 |
| 11 | 75 | 1 | 0 | 0 | 2, 3 | - | - | 0 |
| 12 | 88 | 2 | 0 | 0 | 3 | - | - | 0 |
| 13 | 64 | 1 | 0 | 0 | 2 | - | + | 0 |
| 14 | 71 | 1 | 0 | 0 | 1-2 | + | + | 0 |
| 15 | 62 | 2 | 0 | 0 | 2 | - | - | CMF, Tam |
| 16 | 59 | 2 | 0 | 0 | 2 | - | + | CMF, Tam |
| 17 | 41 | 1 | 0 | 0 | 3 | - | - | CMF |
| 18 | 43 | 1 | 0 | 0 | 0 | - | + | CMF |
| 19 | 43 | 1 | 0 | 0 | 3 | - | + | CMF |
| 20 | 53 | 2 | 0 | x | 3 | - | - | CMF |
| 21 | 60 | 1 | 0 | x | 3 | - | - | CMF |
| 22 | 81 | 1 | 0 | 0 | 3 | + | + | CMF |
| 23 | 72 | 1 | 0 | 0 | 2 | + | - | CMF |
| 24 | 30 | 1c | 0 | 1 | 3 | - | - | CMF |
| 25 | 61 | 1b | 0 | 0 | 3 | - | - | CMF |
| 26 | 67 | 2 | 0 | 1 | 3 | - | - | CMF |
| 27 | 45 | 3 | 0 | 0 | 2 | - | - | CMF, rad |
| 28 | 44 | 2 | 0 | 0 | 2 | + | + | CMF, rad |
| 29 | 86 | 1 | 0 | x | 2, 3 | - | + | CMF, Tam |
| 30 | 41 | 2 | 0 | 0 | 3 | - | - | CMF, Zoladex |
| 31 | 54 | 2 | 0 | 0 | 2 | - | - | EC |
| 32 | 55 | 2 | 0 | 0 | 3 | + | + | EC |
| 33 | 38 | 2 | 0 | 0 | 2 | - | + | EC, rad |
| 34 | 42 | 1 | 0 | 1 | 3 | + | - | EC, rad |
| 35 | 41 | 1 | 0 | 0 | N/D | N/D | N/D | EC, rad, Goserilin, Tam |
| 36 | 56 | 2 | 0 | 0 | 2 | + | + | EC, Tam, rad |
| 37 | 51 | 1 | 0 | 0 | 1 | - | - | FEC |
| 38 | 42 | 1 | 0 | 0 | 2 | + | + | GNRH-Analog |
| 39 | 43 | 1c | 0 | 1 | 2 | - | - | GNRH-Analog |
| 40 | 43 | 1c | 0 | 1 | 2 | - | - | GNRH-Analog |
| 41 | 45 | 1 | 0 | 0 | 2 | + | + | Tam |
| 42 | 53 | 2 | 0 | 0 | 2 | + | + | Tam |
| 43 | 61 | 1a | 0 | 0 | 1 | - | - | Tam |
| 44 | 37 | 2 | 0 | 0 | 3 | - | - | CMF, Tam |
| 45 | 36 | 2 | 0 | 0 | 3 | - | + | CMF, Tam |
| 46 | 53 | 1 | 0 | 0 | 3 | - | + | CMF, Tam |
| 47 | 62 | 2 | 0 | 0 | 3 | + | + | CMF, Tam |
| 48 | 73 | 1 | 0 | x | 2 | - | - | N/A |
| 49 | 64 | 2 | 0 | x | 2 | + | + | N/A |
| 50 | 56 | 2 | 0 | x | 2 | + | - | N/A |
| 51 | 61 | 2 | 0 | x | 2 | + | + | N/A |
| 52 | 69 | 1 | 0 | 0 | 2 | + | + | N/A |
| 53 | 80 | 2 | 0 | 0 | 3 | + | n | N/A |
| 54 | 61 | 2 | 0 | x | 3 | - | - | N/A |
| 55 | 60 | 2 | 0 | x | 2 | - | - | N/A |
| 56 | 72 | 1 | 0 | x | 2 | n | n | N/A |
| 57 | 75 | 2 | 0 | 0 | 2 | + | - | N/A |
| 58 | 60 | 0 | 0 | 0 | N/D | N/D | N/D | N/A |
| 59 | 36 | 2 | 0 | 0 | 3 | + | + | Novaldex |
| 60 | 57 | 1 | 0 | 0 | 1 | + | + | rad |
| 61 | 62 | 2 | 0 | 1 | N/D | N/D | N/D | rad |
| 62 | 53 | 1c | 0 | 0 | 2 | - | + | rad |
| 63 | 62 | 1 | 0 | 0 | 3 | - | - | rad |
| 64 | 47 | 1 | 0 | 0 | 3 | + | + | rad |
| 65 | 48 | 1 | 0 | 0 | 1 | + | + | rad |
| 66 | 44 | 1 | 0 | 0 | N/D | N/D | N/D | rad |
| 67 | 56 | 2 | 0 | 0 | 2 | + | + | rad, Goserelin, 5-Fluoruracil, folic acid |
| 68 | 44 | 1 | 0 | 0 | 2 | - | - | rad |
| 69 | 51 | 2 | 0 | 0 | 3 | - | + | Tam |
| 70 | 50 | 1 | 0 | 0 | 3 | - | + | Tam |
| 71 | 47 | 1 | 0 | 0 | 2 | + | + | Tam |
| 72 | 44 | 2 | 0 | 0 | 2 | - | + | Tam |
| 73 | 44 | 1 | 0 | 0 | 3 | + | + | Tam |
| 74 | 43 | 1 | 0 | 0 | 2 | + | + | Tam |
| 75 | 43 | 1 | 0 | 0 | 2 | + | + | Tam |
| 76 | 48 | 1 | 0 | 0 | 2 | + | + | Tam |
| 77 | 46 | 1 | 0 | 0 | 1 | + | + | Tam |
| 78 | 53 | 1 | 0 | N/A | 2 | + | + | Tam |
| 79 | 53 | 1 | 0 | 0 | 3 | + | + | Tam |
| 80 | 46 | 1 | 0 | 0 | N/A | + | + | Tam |
| 81 | 52 | 1 | 0 | 0 | 2 | + | + | Tam |
| 82 | 42 | 2 | 0 | 0 | 2 | + | + | Tam |
| 83 | 51 | 1 | 0 | 0 | 1 | + | + | Tam |
| 84 | 45 | 1 | 0 | 0 | 2 | + | + | Tam |
| 85 | 39 | 1 | 0 | 0 | 2 | + | + | Tam |
| 86 | 83 | 2 | 0 | x | 2 | + | + | Tam |
| 87 | 66 | 2 | 0 | x | 2, 3 | + | + | Tam |
| 88 | 66 | 2 | 0 | x | 2, 3 | + | + | Tam |
| 89 | 66 | 2 | 0 | x | 2, 3 | + | + | Tam |
| 90 | 65 | 1 | 0 | 0 | 2, 3 | + | + | Tam |
| 91 | 65 | 1 | 0 | 0 | 2, 3 | + | - | Tam |
| 92 | 72 | 2 | 0 | 0 | 2, 3 | + | - | Tam |
| 93 | 60 | 1 | 0 | 0 | 3 | + | + | Tam |
| 94 | 76 | 1 | 0 | 0 | 2 | + | + | Tam |
| 95 | 68 | 2 | 0 | 0 | 2 | + | + | Tam |
| 96 | 67 | 1 | 0 | 0 | 1 | + | + | Tam |
| 97 | 61 | 1 | 0 | -1 | 2, 3 | - | + | Tam |
| 98 | 58 | 1 | 0 | 0 | 2, 3 | - | + | Tam |
| 99 | 72 | 2 | 0 | 0 | 2, 3 | + | + | Tam |
| 100 | 67 | 1 | 0 | 0 | 2 | - | + | Tam |
| 101 | 83 | 2 | 0 | 0 | 2, 3 | - | + | Tam |
| 102 | 48 | 1 | 0 | 0 | N/A | + | + | Tam |
| 103 | 48 | 1 | 0 | 0 | N/A | + | + | Tam |
| 104 | 74 | 1 | 0 | 0 | 2 | + | + | Tam |
| 105 | 59 | 2 | 0 | 1 | 3 | + | - | Tam |
| 106 | 58 | 2 | 0 | 0 | 2 | + | + | Tam |
| 107 | 56 | 1a | 0 | 0 | 2 | - | - | Tam |
| 108 | 53 | 1c | 0 | 0 | 2 | + | + | Tam |
| 109 | 67 | 1b | 0 | 0 | 2 | + | + | Tam |
| 110 | 57 | 1 | 0 | 0 | 2, 3 | - | + | Tam |
| 111 | 62 | 2 | 0 | x | 2 | - | - | Tam |
| 112 | 56 | 1 | 0 | 0 | 3 | + | + | Tam |
| 113 | 72 | 1 | 0 | x | 2 | + | - | Tam |
| 114 | 66 | 1c | 0 | 0 | 2 | + | + | Tam |
| 115 | 57 | 2 | 0 | 0 | 3 | + | + | Tam |
| 116 | 75 | 1 | 0 | 0 | 2 | + | - | Tam |
| 117 | 57 | 1 | 0 | 0 | 2 | + | + | Tam, Aromasin |
| 118 | 80 | 1c | 0 | 0 | 1 | + | - | Tam, rad |
| 119 | 48 | 2 | 0 | 1 | 3 | + | - | Tam, rad CMF, Anastozol |
| 120 | 53 | 2 | 0 | 0 | 2 | + | + | Tam, rad, CMF, Letrozol, |
| 121 | 63 | x | 0 | 1 | N/A | + | - | Tam, rad, Letrozol, Megestrolazetat |
| 122 | 59 | 2 | 0 | 0 | n | + | - | Tam, rad, Letrozol, Megestrolazetat |
| 123 | 62 | 1 | 0 | 0 | 2 | + | - | Tam, EC |
| 124 | 55 | 1 | 0 | 0 | 3 | + | + | Tam, EC, CMF, rad |
| 125 | 41 | 1 | 0 | 1 | 3 | N/D | N/D | Tam, Goserilin, rad |
| 126 | 70 | 2 | 0 | 0 | 3 | + | - | Tam, rad |
| 127 | 68 | 1 | 0 | 1 | 2 | + | - | Tam, rad |
| 128 | 65 | 1 | 0 | 1 | 3 | + | + | Tam, rad |
| 129 | 58 | 2 | 0 | 0 | 3 | - | - | N/A |
| 130 | 48 | 2 | 0 | 0 | N/A | + | + | N/A |
| 131 | 48 | 2 | 0 | 0 | N/A | + | + | N/A |
| 132 | 51 | 2 | 0 | 0 | N/A | N/A | N/A | N/A |
| 133 | 40 | 1 | 0 | 0 | N/A | - | - | N/A |
| 134 | 40 | 1 | 0 | 0 | N/A | - | - | N/A |
| 135 | 47 | 1 | 0 | 0 | N/A | + | + | N/A |
| 136 | 47 | 1 | 0 | 0 | N/A | + | + | N/A |
| 137 | 40 | 2 | 0 | 0 | N/A | - | + | N/A |
| 138 | 83 | 2 | 0 | 0 | N/A | + | + | N/A |
| 139 |  |  |  |  |  |  |  | N/A |
| 140 | 55 | 2 | 0 | 0 | N/A | N/A | N/A | N/A |
| 141 | 56 | 2 | 0 | 0 | N/A | N/A | N/A | N/A |
| 142 |  |  |  |  |  |  |  | N/A |
| 143 | 58 | x | 0 | x |  |  |  | N/A |
| 144 | 85 | x | 0 | x | N/A | N/A | N/A | N/A |
| 145 | N/A | x | 0 | x | N/A | N/A | N/A | N/A |
| 146 | 72 | 2 | 0 | x | 2 | + | - | N/A |
| 147 | 62 | 2 | 0 | x | 3 | + | + | N/A |
| 148 | 62 | 2 | 0 | x | 3 | + | - | N/A |
| 149 | 88 | 2 | 0 | 0 | 1 | - | - | N/A |
| 150 | 83 | 1 | 0 | x | 2 | + | - | N/A |
| 151 | 75 | 2 | 0 | x | 2 | + | + | N/A |
| 152 | 81 | 1 | 0 | 0 | 1 | + | - | N/A |
| 153 | 63 | 0 | 0 | 0 | 1 | + | - | N/A |
| 154 | 63 | 2 | 0 | 1 | 2 | - | - | N/A |
| 155 | 50 | 1 | 0 | x | 3 | - | - | N/A |
| 156 | 63 | 2 | 0 | 0 | 2 | + | + | N/A |
| 157 | 66 | 2 | 0 | 0 | 2 | + | - | N/A |
| 158 | 60 | 1c | 0 | x | 2 | + | - | N/A |
| 159 | 70 | 1c | 0 | 0 | 2 | + | - | N/A |
| 160 | 57 | 1c | 0 | x | 3 | + | + | N/A |
| 161 | 44 | 1c | 0 | x | 3 | + | + | N/A |
